# Carvacrol and Thymol Combat Desiccation Resistance Mechanisms in *Salmonella enterica* Serovar Tennessee

**DOI:** 10.3390/microorganisms10010044

**Published:** 2021-12-26

**Authors:** Ahmed G. Abdelhamid, Ahmed E. Yousef

**Affiliations:** 1 Department of Food Science and Technology, The Ohio State University, 2015 Fyffe Court, Columbus, OH 43210, USA; abdelhamid.9@osu.edu; 2 Botany and Microbiology Department, Faculty of Science, Benha University, Benha 13518, Egypt; 3 Department of Microbiology, The Ohio State University, 105 Biological Sciences Building, 484 West 12th Avenue, Columbus, OH 43210, USA

**Keywords:** natural antimicrobials, *Salmonella enterica*, desiccation resistance, low-a_w_ foods, carvacrol, thymol

## Abstract

Some *Salmonella enterica* serovars are frequently associated with disease outbreaks in low-moisture foods (LMF) due to their ability to adapt efficiently to desiccation stress. These serovars are often persistent during food processing. Disruption of these resistance responses was accomplished previously using the membrane-active lipopeptide, paenibacterin. This study was initiated to determine how desiccation resistance mechanisms are overcome when *Salmonella* Tennessee, a known resistant serovar, is treated with the membrane-active food additives carvacrol and thymol. Knowing that the minimum inhibitory concentrations (MICs) of carvacrol and thymol against *Salmonella* Tennessee are 200 and 100 µg/mL, the concentrations tested were 100–400 and 50–200 µg/mL, respectively. Results show that desiccation-adapted *Salmonella* Tennessee, prepared by air drying at 40% relative humidity and 22–25 °C for 24 h, was not inactivated when exposed for 4.0 h to less than 2xMIC of the two additives. Additionally, treatment of desiccation-adapted *Salmonella* Tennessee for 120 min with carvacrol and thymol at the MIC-level sensitized the cells (1.4–1.5 log CFU/mL reduction) to further desiccation stress. Treating desiccation-adapted *Salmonella* Tennessee with carvacrol and thymol induced leakage of intracellular potassium ions, reduced the biosynthesis of the osmoprotectant trehalose, reduced respiratory activity, decreased ATP production, and caused leakage of intracellular proteins and nucleic acids. Carvacrol, at 200–400 µg/mL, significantly downregulated the transcription of desiccation-related genes (*proV*, STM1494, and *kdpA*) as determined by the reverse-transcription quantitative PCR. The current study revealed some of the mechanisms by which carvacrol and thymol combat desiccation-resistant *Salmonella* Tennessee, raising the feasibility of using these additives to control desiccation-adapted *S. enterica* in LMF.

## 1. Introduction

Some nontyphoidal *Salmonella enterica* serovars are highly adaptable to dry conditions, and this adaptation allows the pathogen to survive for extended periods in low-moisture foods (LMF), and cause frequent disease outbreaks [1]. Dry-tolerant serovars include *Salmonella* Tennessee, Agona, Bredeney, Mbandaka, Montevideo, and Enteritidis, which caused salmonellosis linked to consumption of products such as peanut butter, breakfast cereal, tahini, and almonds. Serovars having this phenotype invoke several mechanisms upon exposure to desiccation conditions; these mechanisms include accumulation of potassium ions, biosynthesis of the osmoprotectant trehalose, increased fatty acid metabolism, and production of cellulose and fimbriae [2]. Adaptability of *Salmonella* serovars to dry conditions was found to involve collateral adaptive responses, such as the ability to form biofilm and induction of viable but nonculturable state [3,4]. Collectively, these responses make desiccation-adapted salmonellae difficult to eliminate by processing, particularly during thermal treatments [5].

Researchers explored the suitability of different methods to combat desiccation-resistant *Salmonella* serovars on dry products such pistachios, nuts, or in-shell pecans; these methods include heat-spray, irradiation, and hot water treatment [6,7,8]. In a previous study, a membrane-active microbial lipopeptide, paenibacterin, was found to disrupt the desiccation resistance in some *S. enterica* serovars; the lipopeptide rendered the pathogen sensitive to dry conditions [9]. However, paenibacterin has not been approved for use in food, and producing the compound in large quantities necessitates laborious purification procedures [10]. Alternatively, membrane-active food ingredients and additives that can combat desiccation-adapted *Salmonella*, in similar fashion to paenibacterin, were investigated [11]. Of these tested compounds, carvacrol and thymol were the most capable of causing the leakage of potassium ions from the cells of selected desiccation-resistant *Salmonella* serovars.

Carvacrol and thymol are monoterpene phenolic compounds that have been studied extensively for their antimicrobial, antitumor, and anti-inflammatory activities [12,13,14,15,16]. The two compounds are used industrially as flavoring food additives [17]. Researchers demonstrated the effect of carvacrol and thymol against bacterial pathogens and presumed that cytoplasmic membrane damage was the mode of antimicrobial action [12]. We hypothesized that carvacrol and thymol are candidate food additives to combat desiccation-resistant *Salmonella*, in view of their activity against bacterial cytoplasmic membranes. In a previous study, the two additives sensitized desiccation-adapted *Salmonella* to heating at 55 °C for 15 min [11]. Furthermore, the anti-*Salmonella* effect of carvacrol was apparent during spray- or freeze-drying of liquid milk, and this effect was noticeable during storage of the dried milk at room temperature [11]. These findings prompted us to explore the mechanisms by which carvacrol and thymol impair desiccation resistance in *Salmonella*. Unveiling such mechanisms will help in confirming the ability of the two compounds to overcome desiccation adaptation in *Salmonella*. This knowledge could facilitate developing membrane-active agents that de-adapt desiccation-resistant *Salmonella*, with the goal of improving the safety of LMF. Therefore, the present study was conducted to investigate the mode of action of membrane-active carvacrol and thymol against the desiccation resistance mechanisms employed by *S. enterica*.

## 2. Materials and Methods

### 2.1. Food Additives

Carvacrol and thymol (96–99% purity; Sigma Aldrich, St. Louis, MO, USA) were dissolved in sterile water containing dimethyl sulfoxide (DMSO; Sigma Aldrich) at 5% level. Control experiments were completed using sterile aqueous DMSO (5%).

### 2.2. Desiccation-Adapted Salmonella

*Salmonella enterica* serovar Tennessee E2007000304, a known desiccation-resistant strain [3,18], was obtained from the food microbiology laboratory culture collection at the Ohio State University and used in this study. This serovar was found previously to survive well during desiccation and subsequent storage in the dry state [9], and to express necessary desiccation-related genes upon exposure to desiccation [3]. Additionally, *Salmonella* Tennessee demonstrated stronger resistance to desiccation stress when compared to *Salmonella* Typhimurium [19], and a genomic association of this serovar with contamination of peanut butter was established in a previous study [20].

**Figure 1:**
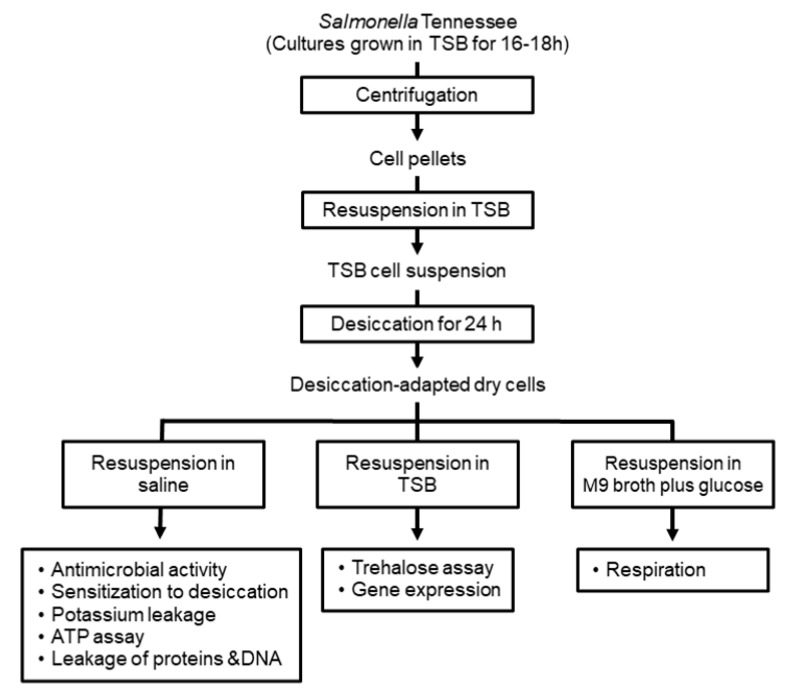
Schematic representation for the preparation of desiccation-adapted cell suspensions implemented in various experiments in the current study.

Desiccation-adapted *Salmonella* Tennessee cells were prepared as described previously [9] with modifications. Briefly, *S. enterica* cells were grown overnight in Tryptic Soy broth (TSB; Becton Dickinson, Sparks, MD, USA) at 37 °C to a cell density of 10^9^ CFU/mL, as determined by plating on Tryptic Soy agar (TSA; Becton Dickinson). Cells of the incubated cultures were harvested by centrifugation at 4 °C and 8000× *g* for 5 min, and finally resuspended in fresh TSB. Aliquots (l mL, each) from cell suspensions were placed in empty plastic Petri dishes (90 mm diameter; VWR International, Radnor, PA, USA) with a final population of 10^9^ CFU per plate, and air dried in a biosafety cabinet for 24 h at 22–25 °C under ca. 40% relative humidity; survivors are considered desiccation-adapted cells. The adapted cells were collected by dispensing 1 mL of the resuspension medium onto the dried cells. The resuspension medium was saline (0.85% NaCl), TSB, or M9 minimal medium supplemented with glucose, depending on the medium required in a given experiment, to obtain desiccation-adapted cell suspension (Figure 1). Resuspension of desiccation-adapted cells in saline solution was suitable in challenge experiments to study resistance of desiccated *Salmonella* cells to membrane-active antimicrobials [9]. TSB or M9 medium with glucose was used in the current study under circumstances where desiccated cells needed to express some metabolic activities [3,4,9].

### 2.3. Antimicrobial Activity of Carvacrol and Thymol against Desiccation-Adapted Salmonella

Cells of desiccation-adapted *Salmonella* Tennessee in saline (Figure 1) were treated with the additives at final concentrations ranging from sublethal (0.5× MIC) to lethal (2× MIC); these correspond to 100, 200, and 400 µg/mL carvacrol, and 50, 100, and 200 µg/mL thymol. Untreated desiccation-adapted cells (0 µg/mL additive) served as a control. The antimicrobial assay was performed in 96-well microtiter plate (Corning, Tewksbury, MA, USA) where each well received 50 µL of the tested compound to produce the desired final concentration, and 50 µL of desiccation-adapted cells in saline. The mixtures were incubated at 22–25 °C for 4.0 h and the surviving populations of *S. enterica* were determined after 0.5, 1.0, 2.0, and 4.0 h of incubation by plating on TSA.

### 2.4. Sensitizing Salmonella to Desiccation Stress by Carvacrol and Thymol

Cells of desiccation-adapted *Salmonella* Tennessee in saline (Figure 1) were treated with carvacrol or thymol at a final concentration of 200 or 100 µg/mL, respectively, and the mixtures were incubated for 120 min at 37 °C to allow the food additives to interact and sensitize the *Salmonella* cells to subsequent desiccation stress. Untreated desiccation-adapted cells in saline served as a control. After the 120-min treatment, cells were harvested by centrifugation at 8000× *g* for 5 min, washed with saline solution twice to remove the food additives, and resuspended in fresh TSB. The surviving populations after the 120-min treatment were determined by plating on TSA. Simultaneously, carvacrol- or thymol-treated and untreated cells were desiccated for an additional 24 h, and population counts were determined to investigate the effect of pre-treatment with additives on sensitizing *Salmonella* Tennessee to desiccation.

### 2.5. Release of Intracellular Potassium Ion from Desiccation-Adapted Salmonella

The potassium-sensitive benzofuran isophthalate probe (PBFI; Invitrogen, Carlsbad, CA, USA) was used to determine the release of potassium ions from desiccation-adapted *Salmonella* Tennessee as described previously [9,11]. Briefly, aliquots (1 mL, each) of desiccation-adapted cells in saline (Figure 1) were harvested by centrifugation at 8000× *g* for 5 min, and cell pellets were resuspended in 5 mM HEPES buffer (Sigma Aldrich) containing 5 mM glucose (Fisher Scientific, Fair Lawn, NJ, USA). Portions of these cell suspensions (90 µL) were added to wells of a black 96-well microplate (Corning) followed by the addition of the PBFI probe at a final concentration of 2 µM. Then, 10 µL of carvacrol or thymol was added to each well to achieve final concentrations of 80 to 800 µg/mL of each compound, and polymyxin (10 µg/mL) served as a positive control. Concentrations of released potassium ions were measured as changes in fluorescence using a microplate reader (PerkinElmer, Wellesley, MA, USA) at excitation and emission wavelengths of 346 and 505 nm, respectively. Fluorescence measurements were captured every 100 s and normalized by subtracting the background noise. 

### 2.6. Effect of Carvacrol and Thymol on Leakage of Intracellular Contents from Desiccation-Adapted Salmonella

*Salmonella* Tennessee desiccation-adapted cells in saline (Figure 1) were treated with the additives at a final concentration of 100–400 µg/mL carvacrol or 50–200 µg/mL thymol and incubated at 37 °C for 2.0 h with shaking at 200 rpm. Additive-untreated, desiccation-adapted cells in saline served as a control. After incubation, cells were harvested by centrifugation at 8000× *g* for 5 min, and cell-free supernatants from treated or untreated cells were used for determination of released protein and nucleic acid. The protein and nucleic acid were measured using a spectrophotometer (NanoVue UV/visible; GE Healthcare, Chicago, IL, USA) at 280 and 260 nm, respectively, and results are expressed as µg/mL. 

### 2.7. Effect of Carvacrol and Thymol on Trehalose Biosynthesis in Desiccation-Adapted Salmonella

*Salmonella* Tennessee desiccation-adapted cells in TSB (Figure 1) were treated with the food additives to final concentrations of 100, 200, and 400 µg/mL carvacrol, or 50, 100, and 200 µg/mL thymol. A non-additive treated desiccation-adapted cell suspension was used as a control. The treated and non-treated cells were desiccated for 12 h to allow trehalose biosynthesis occurring under desiccation conditions in the presence or absence of carvacrol or thymol. After desiccation, additive-treated and non-treated cells were collected and suspended in saline before incubation at 100 °C for 15 min, to lyse the cells and release the intracellular trehalose. After incubation, cell lysates were cooled to 22 °C and cell debris were precipitated by centrifugation at 8000× *g* for 10 min. The trehalose in supernatants of additive-treated and non-treated cells was measured as described in a previous study [9]. Briefly, supernatants were treated with trehalase (Sigma Aldrich), which hydrolyzes trehalose into glucose. Glucose levels were measured using a glucose assay kit (Sigma Aldrich) as described in a previous study [21]. The decrease in trehalose biosynthesis mediated by each treatment was determined in reference to the untreated control.

### 2.8. Effect of Carvacrol and Thymol on Respiratory Activity in Desiccation-Adapted Salmonella

To measure respiratory activity in *Salmonella* Tennessee, triphenyl tetrazolium chloride (TTC; Fisher Scientific), an electron acceptor that indicates dehydrogenase activity, was used as described previously [22]. Briefly, desiccation-adapted cells in M9 minimal medium supplemented with 0.4% glucose (Figure 1) were treated with carvacrol or thymol at a final concentration of 100–400 µg/mL or 50–200 µg/mL, respectively. Untreated desiccation-adapted cells in M9 medium were used as a control. After the preparation of food additive-treated or non-treated cell suspensions, aliquots (150 μL) of these cells were dispensed into 96-well microtiter plates; this was followed by adding 50 μL of filter-sterilized 0.5% TTC solution (wt/vol) and the mixtures were incubated at 37 °C for 22 h. After incubation, the formation of red formazan was measured using a microplate reader (UV max; Molecular Devices, San Jose, CA, USA) at 540 nm and the decrease in respiratory activity in treated cells was calculated in reference to the untreated control.

### 2.9. Effect of Carvacrol and Thymol on ATP Generation in Desiccation-Adapted Salmonella

Desiccation-adapted *Salmonella* Tennessee cells in saline (Figure 1) were treated with carvacrol at final concentration of 100–400 µg/mL or thymol at 50–200 µg/mL for 2.0 h at 37 °C with shaking at 200 rpm. Untreated, desiccation-adapted *Salmonella* Tennessee cells served as a control. After incubation, aliquots (50 µL) from additive-treated and untreated cells were distributed into 96-well, clear-bottom, black microtiter plates (Corning), and the ATP levels were determined using an assay kit (ATPlite1 step assay kit; PerkinElmer) according to the manufacturer’s instructions. The ATP levels were determined by measurement of changes in luminescence using a microplate reader (PerkinElmer). The decrease in ATP levels in treated cells was calculated in reference to the untreated control.

### 2.10. Effect of Carvacrol and Thymol on Expression of Desiccation-Related Genes in Desiccation-Adapted Salmonella

*Salmonella* Tennessee desiccation-adapted cells in TSB (Figure 1) were treated with carvacrol or thymol at final concentrations of 100–400 µg/mL or 50–200 µg/mL, respectively. Untreated, desiccation-adapted cells in TSB served as a control. Treated and untreated mixtures were incubated under desiccation conditions (air drying, and 40% relative humidity) at 22–25 °C for 8 h to enable assessment of the expression of desiccation–related genes under desiccation conditions. After incubation, *Salmonella* cells were collected and harvested by centrifugation at 8000× *g* for 5 min, and cell pellets were used directly for RNA extraction. Total RNA extraction and RNA cleanup, subsequent cDNA synthesis and analysis of the relative expression of desiccation-related genes were performed as described in a previous study [9] using reverse-transcription polymerase chain reaction (RT–qPCR) technique. The expression of four desiccation-related genes (*kdpA*, *proV*, STM1494, and *otsB*) and the reference gene, *gapA*, which encodes glyceraldehyde-3-phosphate dehydrogenase [23], were measured in treated *Salmonella* Tennessee cells and compared to the untreated control cells using the 2^−ΔΔCt^ method [24]. Primer sequences for the investigated genes are listed in Supplementary Table S1.

### 2.11. Statistical Analysis

All experiments were performed in triplicates and each experiment was repeated twice, independently. Each data point was represented as mean ± SD of the two independent repeats. The statistical analysis was performed using a statistical software (GraphPad Prism 9.0.0; GraphPad software, San Diego, CA, USA). Analysis of variance (ANOVA) with Tukey pairwise comparisons were used to determine significant differences between treatment groups or comparing pairs of treatment. Statistical significance was considered at *p* value of <0.05.

## 3. Results

The preparation of desiccation-adapted dry cells of *Salmonella* Tennessee was performed as reported in previous studies [9,11], described in the materials and methods section, and demonstrated in Figure 1. The desiccation-adapted cells were used in various experiments after resuspension in different media as required in each experiment.

### 3.1. Resistance of Desiccation-Adapted Salmonella to Carvacrol and Thymol

**Figure 2:**
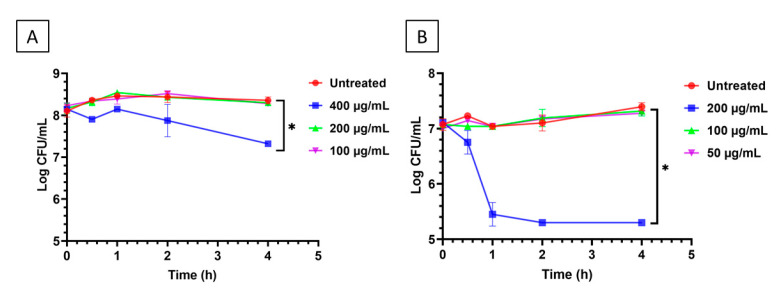
Changes in populations of desiccation-adapted *Salmonella* Tennessee E2007000304 when exposed to different concentrations of carvacrol (panel (**A**)) or thymol (panel (**B**)) for up to 4.0 h at 22–25 °C. Each data point represents mean ± standard deviation. Asterisk (*) denotes significant difference (*p* < 0.05) after 4.0 h of incubation.

The minimum inhibitory concentrations (MICs) of the food additives carvacrol and thymol against non-desiccation-adapted cell suspensions of *Salmonella* Tennessee E2007000304 were determined in a previous study to be 200 and 100 µg/mL, respectively [11]. Based on these findings, the concentrations of carvacrol or thymol tested in the current study against this pathogen were 100, 200, and 400 or 50, 100, and 200 µg/mL, respectively; these concentrations correspond to 0.5×, 1×, and 2× MIC levels, respectively. Desiccation-adapted *Salmonella* Tennessee, suspended in saline (Figure 1), was treated with the additives at 22–25 °C and the surviving populations were determined. When carvacrol was applied at 2× MIC for 0.5–4.0 h, *Salmonella* populations were significantly lower (*p* < 0.05) than those of the untreated control (Figure 2A). Similarly, thymol at 2× MIC resulted in considerable reduction in *Salmonella* populations after 0.5–4.0 h of treatment (*p* < 0.05), when compared to the untreated control (Figure 2B). However, both food additives at 0.5 × and 1× MIC levels did not decrease (*p* > 0.05) *Salmonella* populations when compared to the untreated control during the 4.0-h exposure period. These findings suggest that 2× MIC, at least, of carvacrol or thymol is required to inactivate desiccation-adapted *Salmonella* Tennessee. 

### 3.2. Carvacrol and Thymol Sensitized Salmonella Tennessee to Desiccation Stress

**Figure 3:**
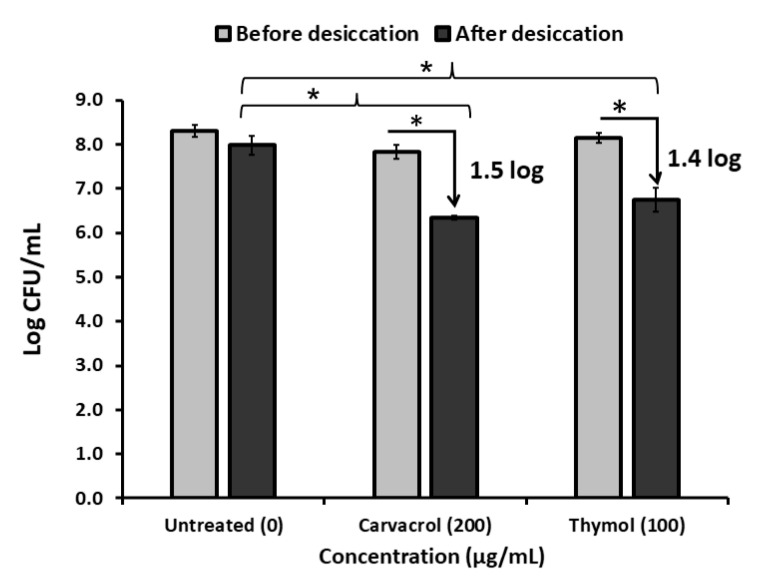
Changes in populations of desiccation-adapted *Salmonella* Tennessee E2007000304 when treated with 200 and 100 µg/mL of carvacrol and thymol, respectively, for 120 min at 37 °C and followed by desiccation for 24 h at 22–25 °C. Each data point represents mean ± standard deviation. Asterisk (*) denotes significant difference at *p* < 0.05.

When desiccation-adapted *Salmonella* Tennessee cells were treated with carvacrol or thymol at 1× MIC level for 120 min and then subjected to a second round of desiccation, the populations behaved differently, compared to the desiccation-adapted additive-untreated control (Figure 3). Results showed that additive-pretreated *Salmonella* Tennessee populations encountered 1.5 or 1.4 log CFU/mL reduction (*p* < 0.05), respectively, compared to the untreated populations, which decreased only by 0.3 log CFU/mL after the second cycle of desiccation. This indicates ability of carvacrol or thymol to sensitize *Salmonella* Tennessee to desiccation stress. 

### 3.3. Carvacrol and Thymol Caused Leakage of Intracellular Potassium Ions from Desiccation-Adapted Salmonella

**Figure 4:**
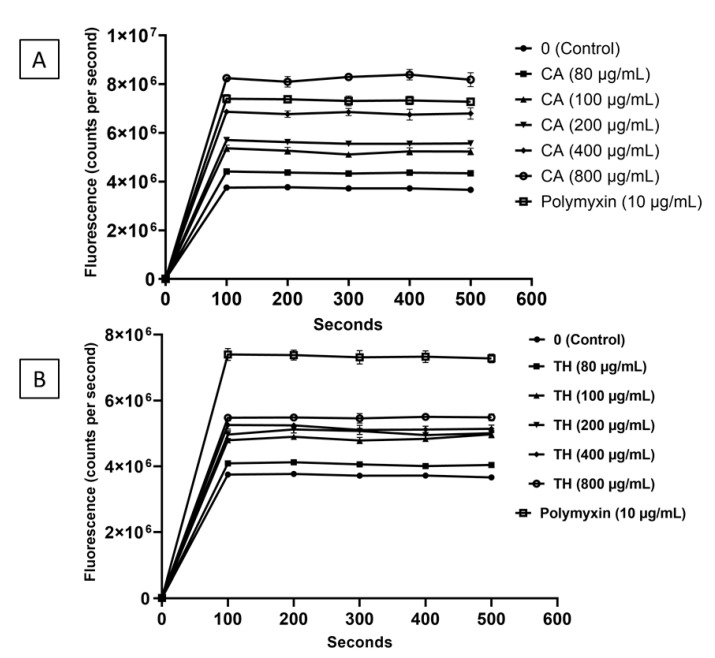
Release of the intracellular potassium ions from desiccation-adapted *Salmonella* Tennessee E2007000304 when treated with different concentrations of carvacrol (panel (**A**)) or thymol (panel (**B**)); polymyxin (10 µg/mL) was used as a positive control. The release of intracellular K^+^ was measured as changes in fluorescence at 505 nm. Each data point represents mean ± standard deviation.

Accumulation of potassium ions (K^+^) is required for *S. enterica* to adapt to desiccation conditions [2], and thus induction of K^+^ leakage could counteract desiccation resistance. Carvacrol or thymol at levels ranging from sublethal to lethal concentrations caused rapid leakage (*p* < 0.05) of potassium ions (after 100 s of exposure to the additive) from desiccation-adapted *Salmonella* Tennessee cells, compared to the untreated desiccation-adapted control (Figure 4). This leakage was most obvious (*p* < 0.05) when the concentration of carvacrol or thymol was >100 or >80 µg/mL, respectively.

### 3.4. Carvacrol and Thymol Caused Leakage of Proteins and Nucleic Acids from Desiccation-Adapted Salmonella Tennessee

**Figure 5:**
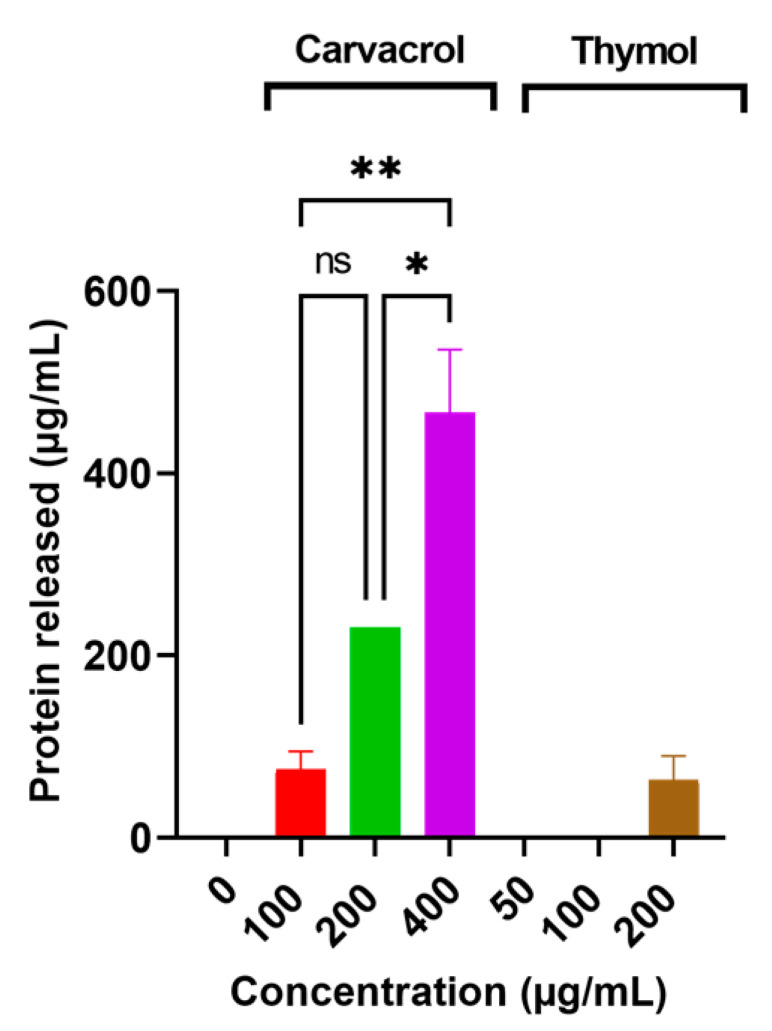
Release of the intracellular protein from desiccation-adapted *Salmonella* Tennessee E2007000304 when treated with different concentrations of carvacrol or thymol for 2.0 h at 37 °C. Each data point represents mean ± standard deviation. Asterisk (*) denotes significant difference at *p* < 0.05; and ** at *p* < 0.01. “ns” denotes non-significant (i.e., *p* > 0.05).

**Figure 6:**
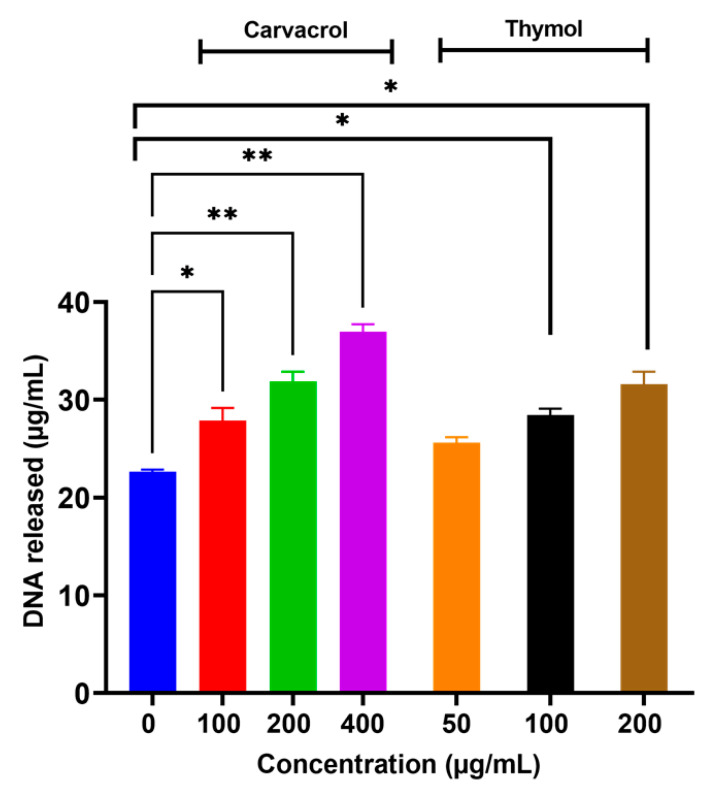
Release of the intracellular nucleic acid from desiccation-adapted *Salmonella* Tennessee E2007000304 when treated with different concentrations of carvacrol or thymol for 2.0 h at 37 °C. Each data point represents mean ± standard deviation. Asterisk (*) denotes significant difference at *p* < 0.05; and ** at *p* < 0.01.

The ability of carvacrol and thymol to induce potassium leakage prompted us to investigate the capability of these additives to invoke the leakage of intracellular macromolecules, such as proteins and nucleic acids. Results showed that carvacrol caused leakage of intracellular proteins in a concentration-dependent manner (*p* < 0.05) as shown in Figure 5. However, thymol at 200 µg/mL only caused leakage of intracellular proteins (Figure 5). Both carvacrol and thymol induced leakage of intracellular nucleic acids in a concentration-dependent manner (*p* < 0.05; Figure 6). There were no sizeable differences (*p* > 0.05) when the two additives were compared for their ability to induce leakage of the intracellular nucleic acids.

### 3.5. Carvacrol and Thymol Decreased Trehalose Biosynthesis in Desiccation-Adapted Salmonella Tennessee

**Figure 7:**
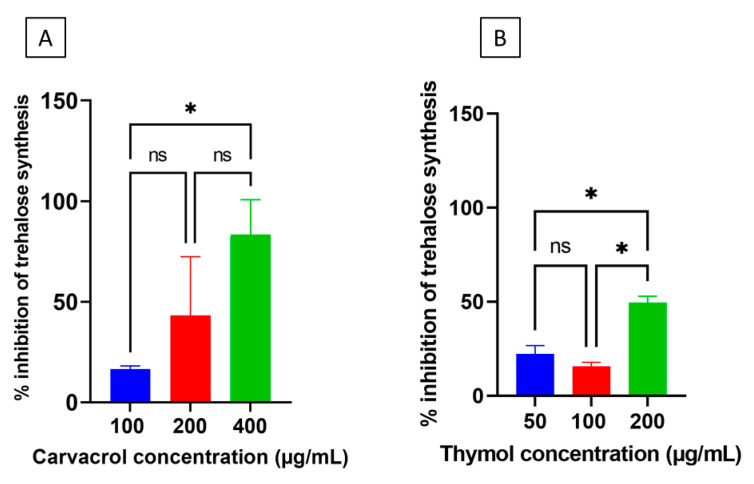
Inhibition of trehalose biosynthesis (per cent) in desiccation-adapted *Salmonella* Tennessee E2007000304 after treatment with different concentrations of carvacrol (panel (**A**)) or thymol (panel (**B**)) for 12 h at 22–25 °C. Each data point represents mean ± standard deviation. Asterisk (*) denotes significant difference at *p* < 0.05. “ns” denotes non-significant (i.e., *p* > 0.05).

The capability of carvacrol and thymol to inhibit trehalose biosynthesis in desiccation-adapted *Salmonella* cells was explored as depicted in Figure 1. Trehalose is an important osmoprotectant that *Salmonella* needs to adapt to desiccation [19]. Figure 7A demonstrated that carvacrol, at all tested concentrations, decreased trehalose levels in desiccation-adapted *Salmonella* cells and this effect was profound at 400 µg/mL of the additive (*p* < 0.05). Thymol caused similar effect at all tested concentrations but the decrease in trehalose levels was significant only when 100 and 200 µg/mL of the compound were tested (Figure 7B). Generally, the inhibitory activity of carvacrol on trehalose biosynthesis was higher than that of thymol (*p* < 0.05). 

### 3.6. Carvacrol and Thymol Decreased Respiratory Activity of Desiccation-Adapted Salmonella Tennessee

**Figure 8:**
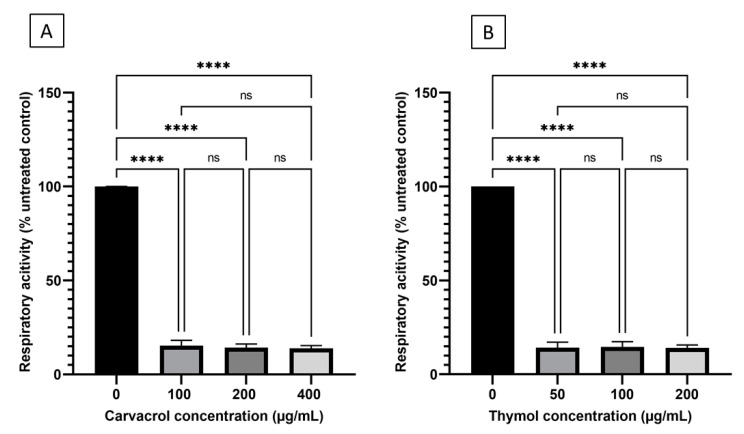
Changes in the respiratory activity of desiccation-adapted *Salmonella* Tennessee E2007000304 after treatment with different concentrations of carvacrol (panel (**A**)) or thymol (panel (**B**)) for 22 h at 37 °C. Each data point represents mean ± standard deviation. Asterisks (****) denote significant difference at *p* < 0.0001. “ns” denotes non-significant (i.e., *p* > 0.05).

*Salmonella* Tennessee desiccation-adapted cells, suspended in M9 broth medium that was supplemented with glucose (Figure 1), were metabolically active in the absence of carvacrol or thymol; however, the cells showed decreased respiratory activity in response to treatment with sublethal to lethal concentrations of the two food additives (Figure 8). *Salmonella* cells lost 75–77% or 76% of their respiratory activity in the presence of carvacrol or thymol, respectively. Despite this trend, there were no significant differences in respiration inhibition (*p* > 0.05) among the various concentrations of each food additive or even between the inhibitory effects of the two additives. 

### 3.7. Carvacrol and Thymol Decreased Adenosine Triphosphate (ATP) Levels in Desiccation-Adapted Salmonella Tennessee 

**Figure 9:**
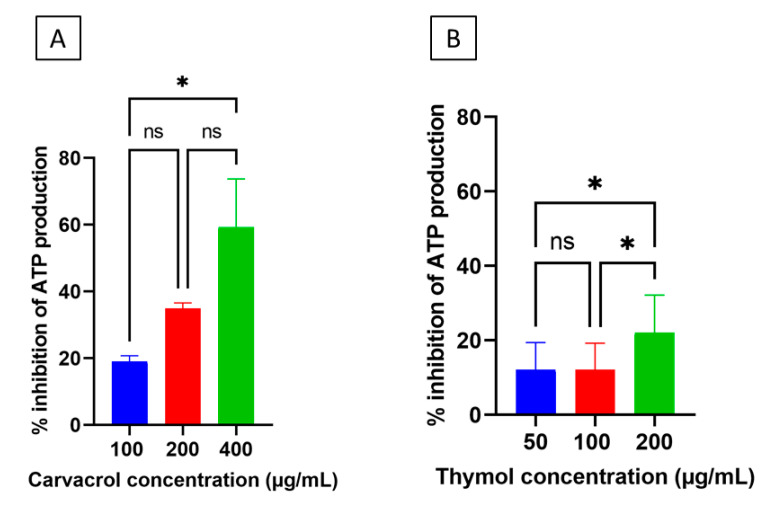
Inhibition of adenosine triphosphate (ATP) production in desiccation-adapted *Salmonella* Tennessee E2007000304 after treatment with different concentrations of carvacrol (panel (**A**)) or thymol (panel (**B**)) for 2.0 h at 37 °C. Each data point represents mean ± standard deviation. Asterisk (*) denotes significant difference at *p* < 0.05. “ns” denotes non-significant (i.e., *p* > 0.05).

Because carvacrol and thymol are membrane-active compounds, and both decreased the respiratory activity in *Salmonella* cells, we explored their ability to affect the production of ATP, which is produced through cytoplasmic membrane- and respiration-related processes [25]. Production of ATP is needed for the import of osmoprotectants or trehalose biosynthesis, processes which are crucial for *Salmonella* to adapt to desiccation [2,19]. Desiccation-adapted *Salmonella* cells suspended in saline (Figure 1) were exposed to carvacrol or thymol at sublethal to lethal concentrations for 120 min, and then the ATP levels were measured in additive-treated and untreated cells. It was obvious that carvacrol reduced ATP levels in a concentration-dependent manner (Figure 9A), with the highest ATP reduction (~60%) achieved when 400 µg/mL of the compound was tested. Thymol generally showed a similar trend (Figure 9B), but the inhibitory effect of carvacrol on ATP generation was higher than that of thymol (*p* < 0.05).

### 3.8. Carvacrol, but Not Thymol, Significantly Altered Expression of Desiccation-Related Genes in Desiccation-Adapted Salmonella Tennessee

The genes investigated in this study (*proV*, *kdpA*, *otsB*, and STM1494) are necessary for desiccation adaptation and are mainly involved in proline accumulation, K^+^ transport, trehalose synthesis, and osmoprotection, respectively [9,19,26]. Expression of these genes was investigated using the RT-qPCR, and results are shown in Table 1. Carvacrol, at 200 µg/mL (i.e., 1× MIC), significantly downregulated (>2-fold decrease) the expression of *proV* and STM1494, whereas a concentration of 400 µg/mL (i.e., 2× MIC) was needed to significantly downregulate (>2-fold decrease) the expression of *proV*, STM1494, and *kdpA* (Table 1). In contrast, thymol at all levels (0.5×–2× MIC) did not cause significant downregulation in the expression of the four genes; this suggests that the anti-desiccation effect of this additive is not related to downregulating the transcription of these genes. 

## 4. Discussion

Despite the efforts of processors to control *S. enterica* in LMF, the bacterium continues to cause frequent outbreaks linked to these foods [27,28]. The desiccation resistance mechanisms help the pathogen to survive for long periods in LMF [29] and cause human illness upon consumption of such contaminated foods. Thermal processing is the most common control method for inactivation of *S. enterica* in LMF, such as almonds, pecans, pistachios, and walnuts, among others [6]. However, desiccation-adapted *Salmonella* expresses thermal resistance [30], which could render heat treatments ineffective in eliminating this pathogen. Additionally, increasing the severity of heat treatment during LMF processing can cause deterioration in product quality and stability [31,32]. 

Several antimicrobials, including oxidizing agents, such as peroxyacetic acid, hydrogen peroxide, and propylene oxide, were used for nonthermal decontamination of LMF. Use of these agents may cause oxidation of food components, which could alter product quality [33]. Although some spices possess antimicrobial activity and potentially provide antimicrobial protection when added as ingredients to LMF, foodborne disease outbreaks linked to spices were frequently reported [34]. These challenges necessitate developing new strategies to combat desiccation resistance in *Salmonella* and to sensitize the pathogen to drying and mild thermal processing. One of these strategies is utilizing cytoplasmic membrane-active agents to de-adapt desiccation-resistant *Salmonella* into a phenotype that is sensitive to drying and thermal processing. The incorporation of membrane-active agents, such as carvacrol and thymol, in liquid foods before drying could be an effective approach to sensitize *Salmonella* to the mild food processing [11].

In a previous study, the microbial-produced lipopeptide paenibacterin disrupted the adaptation mechanisms employed by *Salmonella* Tennessee and Eimsbuettel in response to desiccation [9]. In the current study, we extended this concept to food additives capable of de-adapting the desiccation-resistant *Salmonella* Tennessee E2007000304. Carvacrol and thymol are membrane-active food additives that were found capable of sensitizing desiccation-adapted *S. enterica* to mild heat treatment, and spray- or freeze-drying [11]. In the current study, desiccation-adapted *Salmonella* Tennessee showed tolerance to 1× MIC carvacrol or thymol; these additives were needed at 2× MIC to cause significant reduction in *Salmonella* populations (Figure 2). Similar observations were reported for several desiccation-adapted *S. enterica* serovars in response to treatment with the membrane-active paenibacterin or other chemical antimicrobials [9,35].

The ability of *Salmonella* Tennessee to accumulate K^+^, synthesize trehalose and ATP, retain respiratory activity, and maintain the intracellular contents, is indispensable for desiccation adaptation [2]. Thus, these mechanisms were targeted in the current study when the flavoring food additives, carvacrol and thymol, were tested. Carvacrol and thymol induced rapid leakage of intracellular K^+^ in a concentration-dependent manner at levels ranging from sublethal to lethal concentrations (Figure 4); this effect was comparable to that observed for paenibacterin in a previous study [9]. The induced leakage of intracellular K^+^ may be attributed to the increased cytoplasmic membrane permeability, which can be facilitated by carvacrol or thymol [36]. Potassium efflux could counteract desiccation adaptation, since it is one of *Salmonella*’s early responses to hyperosmotic shock or desiccation conditions [37]. Similar to the K^+^ leakage results, the additives also caused the release of intracellular proteins and nucleic acid (Figure 5 and Figure 6); this confirms the cytoplasmic membrane damage. The bacterial cytoplasmic membrane harbors the essential osmoprotectant transport machinery; hence, it is plausible that membrane changes by the additives adversely impacts desiccation resistance.

The biosynthesis of the trehalose is important for desiccation adaptation in *Salmonella,* since the sugar protects the bacterium against cellular collapse during water loss by forming hydrogen bonding with the membrane phospholipids [38]. Carvacrol and thymol decreased the biosynthesis of the trehalose in *Salmonella* Tennessee (Figure 7). This effect could result in lowering the tolerance of *Salmonella* to desiccation. Evidence for the role of trehalose in desiccation resistance was demonstrated in the finding that desiccation-sensitive *Salmonella* Typhimurium LT2 accumulates inadequate amounts of trehalose [19].

Based on the results of the current study, carvacrol and thymol reduced the respiratory activity in desiccated *Salmonella* Tennessee by more than 70% (Figure 8). A plausible explanation for this observation is that the phenol group in carvacrol or thymol may have acted as a proton exchanger and thus reduced the pH gradient across the bacterial cell membrane [39]; this causes depletion of the proton motif force (PMF) and consequently reduce respiration. Inhibiting ATP production can drastically influence desiccation resistance, since this energy-rich compound is required for the transport of the osmoprotectant molecules into the cell under desiccation stress [2]. The decrease in ATP production, which was observed in the current study (Figure 9), may be attributed to the inactivation of the enzyme ATPase by the hydroxyl group of carvacrol and thymol [33]. 

It was observed that carvacrol at 200–400 µg/mL downregulated the expression of *proV*, STM1494, and *kdpA* (Table 1); these genes are required for importing the osmoprotectant molecules into the cell. This supports the notion that carvacrol can remarkably impair desiccation resistance in *Salmonella.* In contrast, the anti-desiccation effect of thymol could not be attributed to ability of this compound to alter the expression of desiccation-related genes (Table 1). The direct mechanism by which carvacrol impacts expression of desiccation–related genes, particularly those encoding osmoprotectant transporters, has not been entirely explored. However, based on a previous study, treatment of *Salmonella* Enteritidis with carvacrol decreased expression of a panel of cellular proteins that include few transporter proteins [40]. Moreover, essential oils produced by *Origanum vulgare*, which include carvacrol and thymol, were capable of crossing the cytoplasmic membrane of *Salmonella* Enteritidis and acting directly on the bacterial DNA [40]. These observations can explain, in part, the effect of carvacrol on expression of desiccation-related genes. 

**Figure 10:**
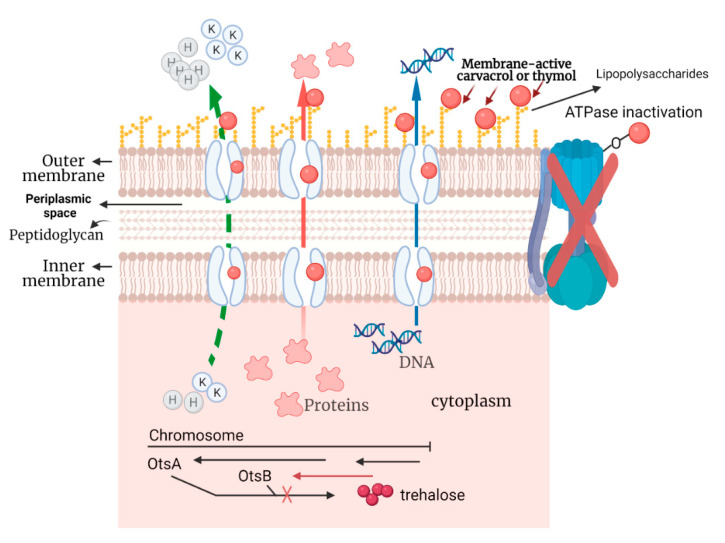
Proposed anti-desiccation resistance mechanisms induced by carvacrol or thymol against desiccation-resistant *Salmonella* Tennessee. The illustration was created using Biorender.com (accessed on 1 December 2021).

The proposed mechanism of action of carvacrol or thymol against desiccation-resistant *Salmonella* is presented schematically in Figure 10. Briefly, the interaction of carvacrol or thymol with the cytoplasmic membrane of desiccation-resistant *Salmonella* Tennessee permeabilizes the cells, causing leakage of the intracellular small ions, such as K^+^, whose intracellular accumulation is needed as a primary event in resistance to desiccation. With the increase in cell-membrane permeability, efflux of other intracellular molecules, such as DNA and proteins, starts. Compromising the cytoplasmic membrane permeability could also alter the proton motive force and reduce respiration. Additionally, the phenol group of carvacrol or thymol may interact with ATPase, causing its inactivation. Several other direct or indirect cascades of events could affect the transcription of genes necessary for desiccation adaption; however, this inference needs future research.

## 5. Conclusions

This study provided molecular evidence that the additives carvacrol and thymol are feasibly usable in mitigating desiccation-resistant *S. enterica* in low–moisture foods. The ability of carvacrol and thymol to act on the cytoplasmic membrane of *Salmonella* Tennessee impaired the desiccation resistance in this pathogen via altering the membrane permeability and inducing the leakage of intracellular osmoprotectants. Considering that *Salmonella* Tennessee has been linked to salmonellosis outbreaks in several low-moisture foods, the current findings imply that carvacrol, thymol, or similar membrane-active agents can protect these foods against this pathogen and potentially against other desiccation-resistant serovars. However, further research is needed to confirm the efficacy of these additives under different desiccation conditions and in various low-moisture foods.

## Figures and Tables

**Table 1:** Relative expression, determined as expression ratio *, of desiccation-related genes in desiccation-adapted *Salmonella* Tennessee, which was treated with carvacrol and thymol and compared to the untreated desiccation-adapted cells.

Desiccation-Related Genes	Carvacrol (µg/mL)			Thymol (µg/mL)		
	100	200	400	50	100	200
*proV*	−1.4 ± 0.19	**−2.1 ± 0.62**	**−2.8 ± 0.91**	−1.2 ± 0.09	1.0 ± 0.07	−1.4 ± 0.19
STM1494	−1.7 ± 0.21	**−2.1 ± 1.4**	**−2.4 ± 2.0**	−1.6 ± 0.63	−1.2 ± 0.18	−1.3 ± 0.27
*kdpA*	−1.4 ± 0.34	0.87 ± 0.61	**−2.1 ± 0.37**	1.5 ± 0.4	1.8 ± 0.42 *	−0.83 ± 0.55
*otsB*	−1.4 ± 0.2	0.43 ± 0.53	−1.4 ± 0.26	0.98 ± 0.02	1.1 ± 0.06	−1.5 ± 0.74

* Values more than +2-fold or less than −2-fold change (bold face), represent significant upregulation or downregulation, respectively.

## Data Availability

Not applicable.

## Supplementary Materials

The following supporting information can be downloaded at: https://www.mdpi.com/article/10.3390/microorganisms10010044/s1, Table S1: Primers used for the reverse transcription-quantitative polymerase chain reaction analysis.
