# Supplementary material for: Carvacrol and Thymol Combat Desiccation Resistance Mechanisms in Salmonella enterica Serovar Tennessee
Source: Microorganisms. 2021 Dec 26;10(1):44. doi: 10.3390/microorganisms10010044 (PMC8779931; doi:10.3390/microorganisms10010044)
Supplement: Supplementary file 1 [file microorganisms-10-00044-s001.zip › microorganisms-1514294-supplementary.pdf]

Supplementary Table S1. Primers used for the reverse transcription-quantitative polymerase chain reaction analysis.

Gene	Forward Primer (5’ –> 3’)	Reverse Primer (5’-> 3’)	References
gapA	GGTGTTGACGTAGTGGCTGAA	AGCGTTGGAAACGATGTCCTG	(1)
proV	CCACAATGGTACGCCTTCTCA	GCATGAGCGCAAATGACTGGA	
STM1494	GCACACCCTCACCCTAAAAC	GCAGGTCGGCTGAGTAAAAT	(2)
KdpA	TGGGACTGGGCATCCTGTT	GGTAGCCGCTGCGGATTTA	(3)
otsB	TTAACCGTATCCCCCGAACTC	CCGCGAGACGGTCTAACAAC	(4)

References

1. Finn S, Händler K, Condell O, Colgan A, Cooney S, McClure P, Amézquita A, Hinton JCD, Fanning S. 2013. ProP is required for the survival of desiccated *Salmonella enterica* serovar Typhimurium cells on a stainless steel surface. Appl Environ Microbiol **79**:4376–4384.

2. Frossard SM, Khan AA, Warrick EC, Gately JM, Hanson AD, Oldham ML, Sanders DA, Csonka LN. 2012. Identification of a third osmoprotectant transport system, the osmU system, in *Salmonella enterica*. J Bacteriol **194**:3861-3871.

3. Gruzdev N, McClelland M, Porwollik S, Ofaim S, Pinto R, Saldinger-Sela S. 2012. Global transcriptional analysis of dehydrated *Salmonella enterica* Serovar Typhimurium. Appl Environ Microbiol **78**:7866–7875.

4. Balaji B, O’Connor K, Lucas JR, Anderson JM, Csonka LN. 2005. Timing of induction of osmotically controlled genes in *Salmonella enterica* serovar Typhimurium, determined with quantitative real-time reverse transcription-PCR. Appl Environ Microbiol **71**:8273–8283.
